# Conditioned Medium from Adipose-Derived Stem Cell Inhibits Jurkat Cell Proliferation through TGF-*β*1 and p38/MAPK Pathway

**DOI:** 10.1155/2019/2107414

**Published:** 2019-12-19

**Authors:** Xiuxia Wang, Yinmin Wang, Xianyu Zhou, Fei Liu

**Affiliations:** ^1^Department of Plastic and Reconstructive Surgery, Shanghai Ninth People's Hospital, Shanghai Jiao Tong University, School of Medicine, Shanghai, China; ^2^Department of Plastic and Reconstructive Surgery, Shanghai General Hospital, Shanghai Jiao Tong University, School of Medicine, Shanghai, China

## Abstract

**Background:**

Since the first report on the immunomodulatory and immunosuppressive properties of Adipose-Derived Stem Cells (ADSCs), many studies have elucidated the underlying molecular mechanism of their suppressive activity on mixed lymphocyte reaction (MLR). However, a gap exists in our understanding of the molecular mechanism of ADSC-conditioned medium (ADSC-CM) on MLR.

**Methods:**

ADSCs were isolated from Human Adipose Tissues, and Enzyme-linked Immunosorbent Assay (ELISA) was used to identify the concentration of transforming growth factor *β*1 (TGF-*β*1) in ADSC-CM. The transcript abundance of TGF-*β*1, as well as that of insulin-like growth factor binding protein 3 (IGF-BP3), was evaluated using qRT-PCR on Jurkat cells cultured in ADSC-CM for 24 hours. The proliferation of the Jurkat cells was assessed using cell cycle assay. Western blotting was performed to identify potential signaling molecules involved in the ADSC-CM-induced inhibition of Jurkat cell proliferation.

**Results:**

The findings confirm that the isolated ADSCs demonstrate classic ADSC characteristics. The level of TGF-*β*1 was found to be low in ADSC-CM, as assessed by ELISA. Jurkat cells grown in ADSC-CM show reduced gene expression of TGF-*β*1 and IGF-BP3 compared with that of the control group. Furthermore, western blotting of ADSC-CM grown Jurkat cells that were blocked at the G0/G1 stage indicates that ADSC-CM decreases the protein expression of pP38 in a dose-dependent manner.

**Conclusion:**

ADSC-CM can inhibit Jurkat cell proliferation through the TGF-*β*1-p38 signaling pathway.

## 1. Introduction

Mesenchymal stem cells (MSCs) are recognized for their immunomodulatory abilities [1–3]. ADSCs, due to their easiness of isolation from an ample tissue source, are a favorable source of MSCs used for the treatment of an extensive variety of diseases [4]. ADSCs do not show the main histocompatibility complex-II expression [5], and their immunosuppressive actions are regulated by prostaglandin E2 (PGE2) [6]. Both preclinical and clinical studies have revealed that allogeneic transplantation of ADSCs is capable of regulating graft-versus-host diseases [7, 8]. Studies have documented that many targeted diseases exhibit significant improvements after ADSC xenotransplantation [9].

Nuclear factor-*κ*B (NF-*κ*B) exists as a constituent of dormant cytoplasmic complexes that are attached to the members of the inhibitor of *κ*B (I*κ*B) family and plays a crucial and evolutionarily role in immunity [10]. Previous studies have shown that ADSCs can modulate T and dendritic cell bioactivity through the NF-*κ*B signaling pathway, in a manner similar to that of the MSCs [11]. Furthermore, paracrine secretions by ADSCs, including cytokines such as IL-10, PGE2, and indoleamine-2,3-dioxygenase (IDO), also have an important role in immunity [6]. Although much research has been focused on the immunosuppressive activity of ADSCs, their effect on the molecular changes during MLR has remained unexplored. Thus, the current study demonstrates the effect of ADSC-CM on Jurkat cells.

## 2. Materials and Methods

### 2.1. Isolation and Cultivation of Human ADSCs

Human adipose tissue samples were acquired from donors who underwent lipoplasty. The acquired adipose tissues were rinsed 3 times using PBS and digested with 0.1% collagenase IV (Roche Diagnostic, Germany) for 1 h. Next, the suspension obtained was centrifuged to obtain the ADSCs. The ADSCs were cultured in Dulbecco's modified Eagle medium (DMEM) comprising 10% fetal bovine serum, 100 U/mL penicillin/streptomycin (Gibco, USA) at 37°C, in a 5% CO_2_ incubator. Once the ADSCs reached 80–90% confluency, they were incubated in DMEM/F12 medium for 24 h. After this incubation, the medium was collected, centrifuged at 300 × *g* for 5 min, and then stored at –80°C. The culture medium of the cells at passages 2–6 was used for the rest of the experiments. Approval was obtained from the Ethics Committee of the School of Medicine at Shanghai Jiao Tong University. Informed consent was obtained from all the patients.

### 2.2. Jurkat Cells

Jurkat cells, purchased from ATCC, were cultured in RPMI medium comprising 10% fetal bovine serum at 37°C in a humidified incubator containing 5% CO_2_.

### 2.3. Flow Cytometry

Following harvesting and washing 3 times in PBS, human ADSCs were incubated with the following FITC-conjugated antibodies: CD29, CD44, CD45, CD90, CD105, CD31, and CD34 (Santa Cruz Biotechnology, USA) at 37°C for 30 minutes in the dark. Flow cytometry was used for detection (BD Biosciences, USA).

### 2.4. Adipogenic and Osteogenic Differentiation

Human ADSCs (10^5^ cells/well) at passages 3–5 were sowed in 0.1% gelatin-coated six-well plates (Cyagen Bioscience, China) and were permitted to achieve 80–90% confluency. Adipogenic differentiation was prompted by growing cells in a medium containing 0.5 *μ*mol/L dexamethasone, 0.5 mmol/L 3-isobutyl-1-methylxanthine, 0.1 mmol/L rosiglitazone, and 100 IU insulin for 2 weeks (Cyagen Bioscience). Osteogenic differentiation was accomplished by growing cells in a medium containing 0.1 *μ*mol/L dexamethasone, 50 *μ*mol/L ascorbic acid, and 10 mmol/L *β*-glycerophosphate for 3 weeks (Cyagen Bioscience). The medium was changed every 3 days. At the end of differentiation, 4% paraformaldehyde in PBS was used to fix the cells for 15 min at room temperature. Next, the cells were stained using Oil Red O and Alizarin Red S, following the supplier's protocol, in order to evaluate adipogenic and osteogenic differentiations, respectively. The number of specifically stained ADSCs was calculated using a light microscope (Olympus, Japan).

### 2.5. Indirect Coculture Conditions

In order to formulate the conditioned medium, human ADSCs from 6 individuals were cultured in DMEM/F12 medium for 24 hours. The cell-free conditioned medium (CM) was centrifuged, carefully removed, and stored at -80°C until further use. The control group of cells was cultured in DMEM/F12 medium for further experiments. Further, 25%, 50%, and 75% cell-free CM in DMEM/F12 were used as the experimental groups.

### 2.6. ELISA

The quantity of TGF-*β*1 in the conditioned medium was determined using an ELISA kit, following the supplier's protocol (R&D Systems, USA).

### 2.7. Quantitative Real-Time Polymerase Chain Reaction (qRT-PCR)

qRT-PCR was performed as previously described [12]. In brief, total RNA was extracted from Jurkat cells following 24 hours of culture with various concentrations of ADSC-CM, using an RNA isolation kit (Takara Bio, Japan). The A260/A280 ratio was used to evaluate RNA purity, which was between 1.8 and 2.0. The following primers were used for gene amplification: TGF-*β*1—forward AAG GAC CTC GGC TGG AAG TG and reverse CCG GGT TAT GCT GGT TGT A; IGF-BP3—forward CCC TCA ACC AAG AAG AAT G and reverse AAC AAG TAG GAC TCC ACC TT. In order to define relative gene expression, the outcomes of 3 autonomous reactions were used. GAPDH expression was used to normalize the findings obtained. Results from three independent experiments each from three technical replicates were used.

### 2.8. Cell Cycle

24 h after growth in ADSC-CM, Jurkat cells were collected and washed once using PBS, then fixed in 70% alcohol, overnight. Cell cycle analysis was conducted using the Cell Cycle Kit (Qihai Biotechnology, China), as per the supplier's protocol. Flow cytometry analysis was conducted using a flow cytometer (Beckman Coulter) and ModiFit LT v2.0 software. Results from three independent experiments each from three technical replicates were used.

### 2.9. Western Blotting

Total protein was extracted from Jurkat cells after 24 or 48 h of coculture with RIPA lysis buffer, as previously published [13]. The nuclear proteins were extracted via NE-PER Nuclear and Cytoplasmic Extraction Reagents (Pierce Biotechnology, Rockford, USA) from the control and experimental groups. Protein samples (40 *μ*g each) were loaded on SDS-polyacrylamide gel, electrophoresed, and then transferred onto PVDF membranes. The protein bands were captured using an enhanced chemiluminescence (ECL) detection kit (Amersham Biosciences, UK). The following primary antibodies were used: phospho-Akt, Akt, phospho-Erk, Erk1/2, phospho-JNK, JNK, phospho-p38, and p38, while GAPDH was used as the loading control. Results from three independent experiments were used.

### 2.10. Statistical Analysis

All data are displayed as the mean ± standard deviation (SD). Statistical analyses were conducted using SPSS version 19.0. Statistical significance was calculated using one-way ANOVA. Post hoc analysis was conducted using Tukey's multiple comparison test. A *p* value of <0.05 was considered to reflect statistical significance.

## 3. Results

### 3.1. Characterization of the ADSCs

**Figure 1:**
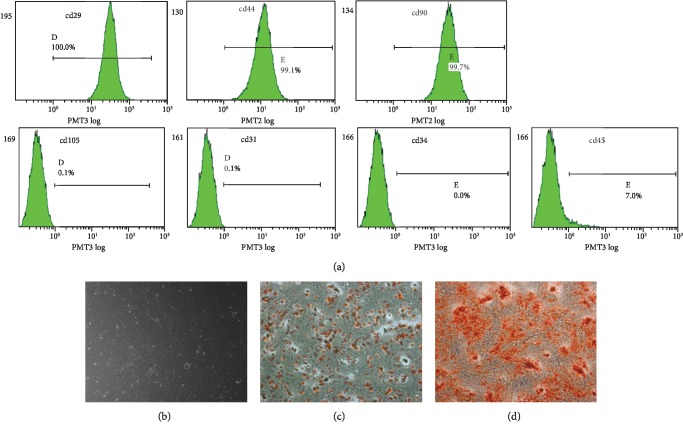
Categorization of human ADSCs. (a) Representation of ADSCs using flow cytometry. ADSCs powerfully express CD29, CD44, CD90, and CD105, but did not express CD31, CD34, or CD45. (b) ADSCs display a fibroblast-like morphology. Cells were promoted to differentiate into adipocytes (c) and osteoblasts (d); bars = 100 *μ*m.

As previously published [14], the human ADSCs indicated positive staining for the following mesenchymal stem cell surface markers: CD29 (100%), CD44 (99.4%), CD105 (85.2%), and CD90 (99.8%), while they indicated negative staining for the following hematopoietic stem cell surface markers: CD31 (0.1%), CD34 (0.1%), and CD45 (0.1%) (Figure 1(a)). The human ADSCs also revealed a classic fibroblast-like morphology (Figure 1(b)). We inspected the multipotent differentiating capability of ADSCs using both adipogenic and osteogenic assays. ADSCs differentiate into an adipogenic phenotype when grown in an adipogenic medium for 2 weeks, as determined through exposure to Oil Red O staining (Figure 1(c)). When cultured for 3 weeks in an osteogenic medium, the ADSCs exhibit mineralization, which was evident after staining with Alizarin Red S. This indicates the existence of calcium deposits (Figure 1(d)). These outcomes indicate that the isolated ADSCs present typical ADSC characteristics.

### 3.2. Scarce TGF-*β*1 Expression in ADSC-CM

**Figure 2:**
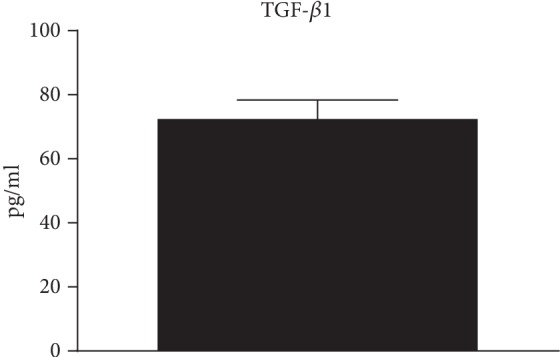
Concentration of TGF-*β*1 in ADSC-CM. ELISA was performed to evaluate TGF-*β*1 levels.

As previous studies have reported, TGF-*β*1 plays a vital role in cell proliferation, growth, and differentiation [15]. Based on our experimental results (Figure 2), the concentration of TGF-*β*1 was found to be only 72.5 ± 5.88 pg/mL, which is too low to have any effect on the cell bioactivity of the secretions from ADSCs into ADSC-CM.

### 3.3. ADSC-CM Decreases the Gene Expression of TGF-*β*1 and IGF-BP3 in Jurkat Cells

**Figure 3:**
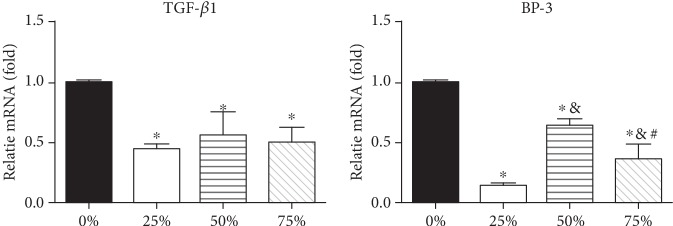
ADSC-CM inhibits gene expression of Jurkat cells. qRT-PCR was used to determine the expression of TGF*β*1 and BP3 in Jurkat cells cultured in the absence or presence of ADSC-CM for 5 days. Gene expression is shown relative to that of control levels (^∗^*p* < 0.05).

IGF-BP3, an IGF binding protein can bind to the TGF-*Β*V receptor (T*β*R-V) and mediate TGF-*β*-induced growth inhibition in concert with the TGF-*β*I receptor (T*β*R-I) and TGF-*β*II receptor (T*β*R-II) [16]. The results of this study indicate that ADSC-CM can suggestively decrease the gene expression of TGF-*β*1 and IGF-BP3 (Figure 3).

### 3.4. ADSC-CM Represses Cell Proliferation

**Figure 4:**
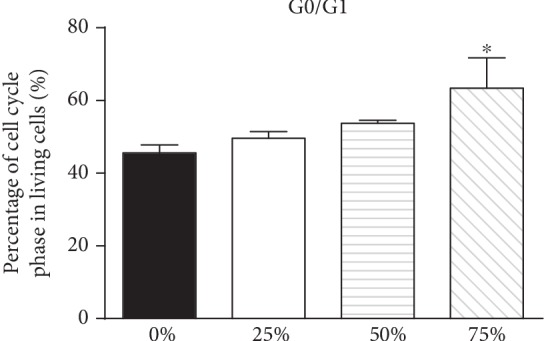
ADSC-CM inhibits Jurkat cell proliferation. The effect of ADSC-CM on the cell cycle profiles was assessed using flow cytometry. (^∗^*p* < 0.05).

Following 24 h of culturing Jurkat cells in the conditioned medium, the number of cells in the G0/G1 phase was found to have increased, along a decrease of cells in the G2/M phase (Figure 4).

### 3.5. ADSC-CM Decreases the Protein Expression of Jurkat Cells

**Figure 5:**
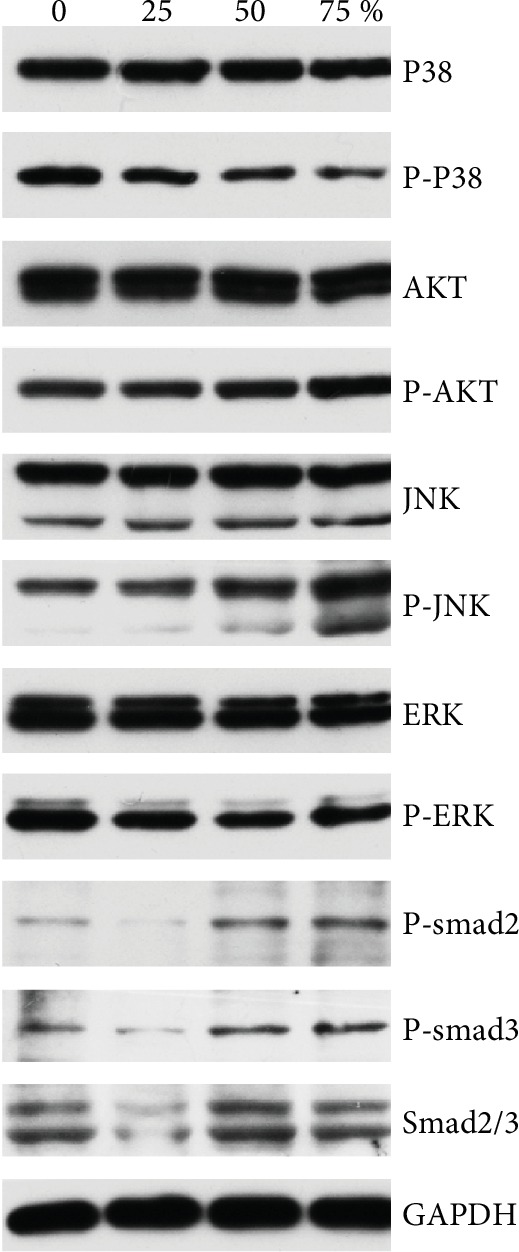
ADSC-CM interferes with intracellular signaling *in vitro*. In order to understand the fundamental mechanism of the antiproliferative effect of ADSC-CM, protein levels in Jurkat cells were determined using western blotting.

The phosphorylation levels of p38 MAPK decreased moderately after culture with Jurkat cells, at different concentrations of ADSC-CM. However, no noteworthy effect on the stimulation of Akt, ERK1/2, and JNK was observed (Figure 5).

## 4. Discussion

The restoration or substitution of injured organs remains to be an imperative and perplexing community health issue. Organ grafting may be the most effective method of curing defective or damaged organs. However, graft rejection continues to be the biggest obstacle for successful and permanent transplantation [17]. There have been a substantial number of research studies that have concentrated on the approach of enduring organ graft transplantations and attenuation of immune rejections. The role of lymphocytes, especially T cells, in graft rejection is vigorous, as demonstrated by experiments on nude mice; these mice did not dismiss allogeneic skin grafts since they were deficient in CD4+ as well as CD8+ functionality [18]. Nevertheless, reestablishing this functionality using adoptive relocation of regular T cells triggers the dismissal of the skin graft [18]. Hence, a difficulty in allogeneic grafting is the activating factor of CD4+ and CD8+ T cell immune responses [19, 20]. Dendritic cells (DCs), macrophages, polymorphonuclear cells, angiogenic mediators, and cytokines also stimulate dismissal [21]. Many immunosuppressive agents that are used to suppress graft rejection cannot effectively inhibit immune responses and elicit many side effects [22].

Scientists have tried to identify an effective way to suppress immune reaction over many years. Since the discovery of MSCs, studies have found that MSCs can move to the location of tissue damage, induce inflammation, and exert an anti-inflammatory effect by regulating dendritic cells, natural killer cells (NK cells), T cells, and B cells [23]. MSCs can also promote regulatory T (Treg) cells and preserve their function [24]. ADSCs, which share the same biological properties with MSC, are promising MSCs for combating immune rejection. The first report on immunomodulatory, as well as the immunosuppressive properties of ADSCs, was published in 2005; to be precise, *in vitro* experimentations have indicated that ADSCs do not aggravate alloreactivity and that they are capable of repressing MLR [25]. Since numerous studies have revealed the prominence of soluble factors, amongst which the utmost recurrently recognized is PGE2, it is believed that the immunosuppressive effects of ADSCs may not require cell-cell contact [26]. Additionally, precise prevention of indoleamine 2,3-dioxygenase or nullification of leukemia inhibitory factor has also been found to eliminate immunosuppression caused by ADSCs [27, 28]. Further, we attempted to find out the mechanism by which the lymphocyte reaction leads to the release of secretomes of ADSCs.

The findings of the present study identified that ADSCs contain CD29, CD44, CD105, and CD90 markers. At the same time, isolated human ADSCs were found to exhibit multilineage differentiation potential and self-renewal ability. Their fibroblast-like morphology was in accordance with that of previous reports [29].

TGF-*β*1 plays a crucial role in cell proliferation and growth. In order to exclude the effect of TGF-*β*1, we used ELISA to detect the concentration of TGF-*β*1 in ADSC-CM. Furthermore, studies show that TGF-*β*1 does not play a pivotal role in the suppression mediated by the passaged ADSCs [25]. As shown in Figure 2, the concentration of TGF-*β*1 in the ADSC-CM was found to be extremely low (75 ± 5 pg/mL); thus, it could not have interfered with the results of subsequent experiments. T*β*R-V serves as the IGF-BP3 receptor that mediates IGF-independent growth inhibition caused by IGF-BP3 in responsive cells. Our results indicate that the gene expression of TGF-*β*1 and IGF-BP3 in Jurkat cells grown in ADSC-CM decreases significantly (Figure 3).

Cell cycle assay was also used to assess cell proliferation and growth conditions. The results of the assay indicate that ADSC-CM-treated Jurkat cells are stuck at the G0/G1 stage of the cell cycle, which corresponds with the RT-PCR results to a certain extent.

Along with analyzing proliferation and growth, we also wanted to identify the possible mechanism involved. Hence, western blotting was used to analyze possible mechanisms underlying the antiproliferative effect of Jurkat cells. Our results reveal that the level of pP38 decreases significantly as the concentration of ADSC-CM increases, indicating that phosphorylation of p38 is important in ADSC-CM-mediated immunosuppression. However, the depletion of culture medium components in ADSC-conditioned medium may influence the results. Thus, further studies to explore the certain molecular effect of the proliferation of T cells in CM remain to be resolved.

Organ graft dismissal is an imperative issue in regenerative medicine. The refusal procedure takes place due to stimulation of T cells, either due to the direct, indirect, or semidirect alloantigen recognition cascades, along with the vigorous involvement of accessory B and NK cells, which abolish donor cells. Efforts to induce tolerance, as well as prolong the endurance of transplants, have not been fully successful. Supplementary alternative approaches, including stem cell-based therapies, are known to be more effective. Treatment with stem cells has an abundant potential in the field of bioengineering, due to the ease of obtaining cells for restoration or even replacement of injured tissue or organs. Hence, ADSCs, which are very similar to MSCs, are easy to obtain and show immense therapeutic potential for repairing damaged tissues.

## 5. Conclusion

To conclude, the results of the current study have revealed that ADSC-CM is able to inhibit Jurkat cell proliferation through the TGF-*β*1/p38 signaling pathway. However, more functional measurements are required before ADSC-CM can be used as a tolerogenic strategy in the near future.

## Data Availability

The flow cytometry, PCR, ELISA, and WB data used to support the findings of this study are included within the article. If there is any other information needed, the corresponding author would provide the information on request.

## References

[1] Devine S. M., Bartholomew A. M., Mahmud N. (2001). Mesenchymal stem cells are capable of homing to the bone marrow of non-human primates following systemic infusion. *Experimental Hematology*.

[2] Liechty K. W., MacKenzie T. C., Shaaban A. F. (2000). Human mesenchymal stem cells engraft and demonstrate site-specific differentiation after *in utero* transplantation in sheep. *Nature Medicine*.

[3] Bartholomew A., Sturgeon C., Siatskas M. (2002). Mesenchymal stem cells suppress lymphocyte proliferation in vitro and prolong skin graft survival in vivo. *Experimental Hematology*.

[4] Sheykhhasan M., Qomi R. T., Ghiasi M. (2015). Fibrin scaffolds designing in order to human adipose-derived mesenchymal stem cells differentiation to chondrocytes in the presence of TGF-*β*3. *International Journal of Stem Cells*.

[5] Niemeyer P., Kornacker M., Mehlhorn A. (2007). Comparison of immunological properties of bone marrow stromal cells and adipose tissue-derived stem cells before and after osteogenic differentiation *in vitro*. *Tissue Engineering*.

[6] Cui L., Yin S., Liu W., Li N., Zhang W., Cao Y. (2007). Expanded adipose-derived stem cells suppress mixed lymphocyte reaction by secretion of prostaglandin E2. *Tissue Engineering*.

[7] Gautam M., Fujita D., Kimura K. (2015). Transplantation of adipose tissue-derived stem cells improves cardiac contractile function and electrical stability in a rat myocardial infarction model. *Journal of Molecular and Cellular Cardiology*.

[8] Gao W., Zhang L., Zhang Y., Sun C., Chen X., Wang Y. (2017). Adipose-derived mesenchymal stem cells promote liver regeneration and suppress rejection in small-for-size liver allograft. *Transplant Immunology*.

[9] Ren M. L., Peng W., Yang Z. L. (2012). Allogeneic adipose-derived stem cells with low immunogenicity constructing tissue-engineered bone for repairing bone defects in pigs. *Cell Transplantation*.

[10] Durand J. K., Baldwin A. S. (2017). Targeting IKK and NF-*κ*B for therapy. *Advances in Protein Chemistry and Structural Biology*.

[11] Sun S. C. (2017). The non-canonical NF-*κ*B pathway in immunity and inflammation. *Nature Reviews Immunology*.

[12] Wang Y., Wang X., Zhou X., Zhu Z., Yang J., Liu F. (2019). Suppressive effect mediated by human adipose-derived stem cells on T cells involves the activation of JNK. *International Journal of Molecular Medicine*.

[13] Wang W., Qu M., Xu L. (2016). Sorafenib exerts an anti-keloid activity by antagonizing TGF-*β*/Smad and MAPK/ERK signaling pathways. *Journal of Molecular Medicine*.

[14] Wankhade U. D., Shen M., Kolhe R., Fulzele S. (2016). Advances in adipose-derived stem cells isolation, characterization, and application in regenerative tissue engineering. *Stem Cells International*.

[15] Dennler S., Goumans M. J., ten Dijke P. (2002). Transforming growth factor beta signal transduction. *Journal of Leukocyte Biology*.

[16] Huang S. S., Huang J. S. (2005). TGF-*β* control of cell proliferation. *Journal of Cellular Biochemistry*.

[17] Papp G., Boros P., Nakken B., Szodoray P., Zeher M. (2017). Regulatory immune cells and functions in autoimmunity and transplantation immunology. *Autoimmunity Reviews*.

[18] Sundberg J. P., Dunstan R. W., Beamer W. G., Roop D. R. (1994). Full-thickness skin grafts from flaky skin mice to nude mice: maintenance of the psoriasiform phenotype. *The Journal of Investigative Dermatology*.

[19] Bradley J. A., Sarawar S. R., Porteous C. (1992). Allograft rejection in CD4+ T cell-reconstituted athymic nude rats—the nonessential role of host-derived CD8+ cells. *Transplantation*.

[20] Wood K. J., Goto R. (2012). Mechanisms of rejection: current perspectives. *Transplantation*.

[21] Richters C. D., van Pelt A., van Geldrop E. (1996). Migration of rat skin dendritic cells. *Journal of Leukocyte Biology*.

[22] Peng X., Luo X., Hou J. Y. (2017). Immunosuppressive agents for the treatment of primary sclerosing cholangitis: a systematic review and meta-analysis. *Digestive Diseases*.

[23] Cho K. A., Lee J. K., Kim Y. H., Park M., Woo S. Y., Ryu K. H. (2017). Mesenchymal stem cells ameliorate B-cell-mediated immune responses and increase IL-10-expressing regulatory B cells in an EBI3-dependent manner. *Cellular & Molecular Immunology*.

[24] Tang R. J., Shen S. N., Zhao X. Y. (2015). Mesenchymal stem cells-regulated Treg cells suppress colitis-associated colorectal cancer. *Stem Cell Research & Therapy*.

[25] Puissant B., Barreau C., Bourin P. (2005). Immunomodulatory effect of human adipose tissue-derived adult stem cells: comparison with bone marrow mesenchymal stem cells. *British Journal of Haematology*.

[26] Wolbank S., Peterbauer A., Fahrner M. (2007). Dose-dependent immunomodulatory effect of human stem cells from amniotic membrane: a comparison with human mesenchymal stem cells from adipose tissue. *Tissue Engineering*.

[27] Kang J. W., Kang K. S., Koo H. C., Park J. R., Choi E. W., Park Y. H. (2008). Soluble factors-mediated immunomodulatory effects of canine adipose tissue-derived mesenchymal stem cells. *Stem Cells and Development*.

[28] Najar M., Raicevic G., Boufker H. I. (2010). Adipose-tissue-derived and Wharton’s jelly-derived mesenchymal stromal cells suppress lymphocyte responses by secreting leukemia inhibitory factor. *Tissue Engineering Part A*.

[29] Li Y., Zhang W., Gao J. (2016). Adipose tissue-derived stem cells suppress hypertrophic scar fibrosis via the p38/MAPK signaling pathway. *Stem Cell Research & Therapy*.

